# Identification of the pore-forming and binding domains of the *Sneathia vaginalis* cytopathogenic toxin A

**DOI:** 10.1371/journal.pone.0284349

**Published:** 2023-05-04

**Authors:** Cathyrn K. O’Brien, Jacob R. Raskin, Ivypel Amankwa Asare, Christine Wei, Joy Ma, Zion T. McCoy, Kimberly K. Jefferson

**Affiliations:** 1 Department of Microbiology and Immunology, Virginia Commonwealth University, Richmond, Virginia, United States of America; 2 Department of Biomedical Sciences University of Lynchburg, Lynchburg, Virginia, United States of America; 3 Department of Obstetrics and Gynecology, Virginia Commonwealth University, Richmond, Virginia, United States of America; University of Illinois Urbana-Champaign, UNITED STATES

## Abstract

The association between *Sneathia vaginalis* and preterm birth is emerging. The Gram-negative anaerobe produces a large exotoxin, the cytopathogenic toxin A (CptA), that forms pores in human epithelial cells and red blood cells. The structure of the toxin has not been determined, but in silico analysis predicts that a large amino-terminal region of the protein is globular and separated from the carboxy-terminal tandem repeats by a disordered region. We found that a recombinant protein consisting of the predicted structured amino-terminal portion of CptA and devoid of the repeat region was sufficient to permeabilize epithelial cells and red blood cells. The repeat region was capable of binding to epithelial cells but did not permeabilize them or lyse red blood cells. CptA is the only *S*. *vaginalis* virulence factor that has been examined mechanistically to date, and this analysis sets the foundation for an understanding of how this novel pore-forming toxin exerts its activity.

## Introduction

Bacteria in the genus *Sneathia*, which currently includes two named species, *vaginalis* and *sanguinigens*, have undergone multiple taxonomic realignments. The Gram-negative, rod-shaped anaerobes were formerly categorized in the genus *Leptotrichia*, but were found to be genetically and phenotypically distinct and were assigned to a new genus [1]. Our lab isolated and sequenced the genome of the type strain for one of the species within this genus and we named it *S*. *amnii* due to its association with amnionitis [2] and to honor its original designation of *Leptotrichia amnionii*. However, it was later renamed *S*. *vaginalis* because the type strain that we isolated was from a vaginal swab sample [2].

*Sneathia* species have been linked to preterm birth and other obstetric and gynecologic clinical problems [3–8]. Importantly, we and others have found that *Sneathia* is significantly more abundant in the vaginal microbiome of Hispanic females and those of African ancestry; groups who are also disproportionately affected by preterm birth (PTB) and bacterial vaginosis (BV) [9–11]. A study by Nelson et al. found that in women who had experienced a PTB in a prior pregnancy, the adjusted risk for spontaneous PTB in their present pregnancy was more than 9-fold greater if their vaginal microbiomes contained increasing levels of *Sneathia* over the first 24 weeks of pregnancy [12]. A recent study by Dr. Roberto Romero et. al., found that *S*. *vaginalis* was the most commonly identified infectious agent in the amniotic fluid (28.5%) of subjects with preterm premature rupture of membranes (PPROM) [13]. A study of preeclampsia also found that when bacteria were detected in amniotic fluid, *Sneathia* was the most common taxon identified (50% of cases where infection was present) [7]. Furthermore, in a study by DiGiulio et. al. that used PCR to detect a panel of potentially pathogenic bacterial species in amniotic fluid, a strong association was found between *Sneathia* species and preterm birth and (note that two of the taxa that they detected were annotated differently at the time of publication, but they are now categorized as *Sneathia*) [5].

Outside of pregnancy, *Sneathia* spp. have been implicated in a number of infections in both the male and female genitourinary tract. The link between HIV transmission and BV has been well documented [14–16] and recently, *Sneathia*, along with other BV species, were found to be specifically associated, through 16S gene survey analyses, with an inflammatory vaginal milieu and increased HIV transmission [17]. Women from whom *Sneathia* can be detected in endometrial biopsies by PCR are more likely to experience recurrent pelvic inflammatory disease and infertility [18]. *S*. *vaginalis* has been identified by culture and 16S sequencing in pus aspirates from a case of tubo-ovarian abscess [19] and in multiple cases of salpingitis [20]. Reports have demonstrated that *Sneathia* often goes undetected by traditional methods of bacterial identification [7,21,22]. The organism is fastidious as it requires human blood components and anaerobic conditions, and methods of identification in the setting of the clinical microbiology laboratory are inadequate. Thus, it evades detection by clinical labs and this likely explains why it has only recently begun to emerge as an important pathogen of the female reproductive tract.

To date, very little is known about the biology and virulence of *S*. *vaginalis*. From previous research in our lab, an exotoxin, cytopathogenic toxin A (CptA), was identified and found to possess both hemolytic and cytotoxic activity [23]. In this study, antiserum-mediated inhibition of CptA activity prevented *S*. *vaginalis* from traversing human chorion in vitro [23]. CptA is the effector component of a Type Vb (two partner) secretion system. Type Vb secretion system effectors are typically large, multi-domain proteins that produce a virulent effect on the host [24]. CptA is a large toxin, predicted to be 226 kDa, and structural prediction suggests that it may have a multi-domain structure. Bacterial pore-forming toxins composed of multiple, separable domains are common. Repeats in toxin, (RTX) pore-forming toxins, have a catalytic domain and a C-terminal calcium-binding domain composed of a series of 9 amino acid tandem repeats that are rich in glycine and aspartate residues [25]. A subfamily of the RTX toxins, the multifunctional autoprocessing repeats-in-toxin (MARTX) toxins have autoproteolytic activity that separates the domains and activates the toxin function [26]. The cholesterol-dependent cytolysin family of pore-forming toxins are composed of distinct domains involved in binding and pore-formation and chimeric toxins have been assembled by combining domains from different members of this toxin family [27]. The carboxyl-terminal end of CptA contains a series of seven tandem repeats, each 83 amino acids in length, suggesting that CptA may also have a multi-domain structure. In this study, we initiated an investigation to determine whether the domains of CptA are separable, and found evidence that the repeat region is capable of binding to human epithelial cells while the amino terminal portion is necessary and sufficient for the pore-forming activity of the toxin.

## Materials & methods

### Bacterial strains, cell line and growth conditions

*S*. *vaginalis* strain Sn35 was cultured anaerobically in Porcine Brain Heart Infusion (BHI) broth containing 5% fetal bovine serum (FBS). *E*. *coli* strains were grown aerobically in Luria Bertani containing 100 μg/mL ampicillin at 37°C. *E*. *coli* strain Stable (New England Biolabs) was used for plasmid isolation and maintenance and strain BL21 (DE3) pLysS + pRIPL was used for recombinant protein expression. Jeg-3 (ATCC HTB-36) chorionic trophoblast cells were cultured in Eagle’s Modified Essential Medium (EMEM) (Quality Biological) supplemented with 10% FBS and 1 IU mL^-1^ penicillin/streptomycin (pen/strep) at 37°C in 5% CO_2_.

### Production of recombinant truncated CptA proteins

The *cptA* gene lacking the portion predicted to encode the signal peptide, CptA24-2130 was amplified by PCR using the primer pair PFD1FWD and RDREV (Table 1), CptA24-1349 was amplified using was amplified using PFD1FWD and PFD1349REV, and CptA1342-2130 was amplified using Repeats4FWD and RDREV and Phire Hot Start II polymerase (Thermo). The amplicons were purified from agarose gels using the Qiaquick Gel Extraction kit (Qiagen), digested with BamHI and XhoI (New England Biolabs) and ligated to the pET32a vector using Quick-stick ligase master mix (Bioline). The pET32a vector adds a TrxA-tag, a 6-histidine tag, an S-tag, and enterokinase and thrombin cleavage sites. Together, the tags add ~19kDa to the amino terminus of expressed proteins and were left intact as this was shown in our previous study to improve stability without affecting toxin activity [23]. Cloning and plasmid maintenance was in Stable Competent cells from New England Biolabs. The plasmid was transformed into *E*. *coli* BL21-CodonPlus (DE3)-RIL cells (Agilent) for expression. Bacteria were grown in LB containing 100 μg ampicillin and 50 μg chloramphenicol per mL at 37°C and expression was induced, when the OD_595nm_ reached 0.5, for 2 hours with 1mM IPTG at 21°C with shaking at 225 rpm. Bacteria were collected and lysed using a French press in 50 mM NaH_2_PO_4_, pH 8.0 containing 300 mM NaCl, EDTA-free COMPLETE protease inhibitors (Roche), 5mM EDTA, and 0.1% Triton X-100. Insoluble material was removed from the lysate by centrifugation at 30,000 X g and the proteins were purified by histidine affinity using cOmplete™ His-Tag Purification Resin (Sigma-Aldrich) according to manufacturer’s instructions and eluted with 200 mM imidazole. The fractions were concentrated to approximately 4–5 mg/ml using 50,000 da molecular weight cutoff Amicon Ultra-0.5 centrifugal filters and 0.5 ml was loaded onto a Superose 6 Increase 10/300 GL within an AKTA Pure FPLC system. The system collected 0.5mL fractions and all fractions were tested for hemolytic activity and analyzed by SDS PAGE. Fractions containing proteins of the correct size were pooled and concentrated and stored at -80C in 50 mM NaH_2_PO_4_, pH 8.0 containing 300 mM NaCl and 5% glycerol.

**Table 1:** Primers used in this study.

Primer name	Sequence (5’-3’)
PFD1FWD	gcgaatcggatccAAAACAATAGTAGACACTACTAAAAGTAAC
PFD1349REV	gcgaatctctcgagGTTTAGGATTTACAAGTTTACCGTC
Repeat4FWD	gcgaatcggatccGACGGTAAACTTGTAAATCCTAAAC
RDREV	gcgaatctctcgagTTATCTATGTCTTCTTCTAAATCTAGATAGTAC

### Immunofluorescence staining

Purified CptA24-2130, CptA24-1349, and CptA1342-2130 were labelled with Alexa488 using the Molecular Probes™ Fluorescent Protein Labeling Kit according to manufacturer’s instructions. Jeg-3 cell monolayers were cultured in 35 mm glass-bottom dishes (MatTek) to confluence. The cells were washed once with PBS, and 1mL fresh media containing 100 nM fluorescently labeled CptA24-2130, CptA24-1349, or CptA1342-2130, or 25uL PBS (vehicle control) was added to each dish. After 1 hr at 37°C 5% CO_2_, 5mL Vybrant™ DiL cell membrane stain was added to each dish. After a further 15 min, non-adherent proteins were removed by flooding the dishes 5 times with PBS and 1mL PBS was added to each dish. The monolayers were visualized using an EVOS M5000 and a 40X objective. Each protein was tested in three separate biologic replicates. Images in Tif format were uploaded to ImageJ [28] and minimum and maximum pixel intensities were adjusted consistently for all samples to enhance on-screen viewing (green 0–100, blue 0–150, trans 25–150). For quantitative analysis of fluorescent protein binding, Jeg-3 cells were cultured in 24 well plates. After the monolayers reached confluence, the media was removed and replaced with 200uL fresh media containing 10uL PBS, or 400 nM, 200 nM, or 100 nM fluorescently labeled CptA24-2130, CptA24-1349, or CptA1342-2130. The cells were protected from light and incubated for 1.5 hr at 37°C 5% CO_2_. The media was collected and placed in a flat-bottom black well, black bottom 96 well plate while the monolayers were washed 3 times with 1 mL PBS. The monolayers were carefully scraped from the bottom of the wells into 100 uL PBS and placed in the black well 96 well plate. Fluorescence was assessed using a BioTek Synergy HT with excitation 485+/-20nm and emission 525+/-20nm and sensitivity level 100.

### Hemolysis

Human whole blood collected in EDTA was purchased from BioChemed Services (Winchester, VA). Red blood cells (RBCs) were prepared by centrifuging blood at 500* × g* for 8 min and washing with PBS three times. RBCs were resuspended in 3X the original blood volume of PBS and ~10^5^ RBC in 100 mL was added to wells of a 96 well plate. The recombinant proteins were incubated with the RBCs at 37^○^ for 2 or 12 hr. Following incubation, the plate was gently agitated to release hemoglobin from lysed RBCs, intact RBCs were collected by centrifugation at 500* × g* for 8 min, the supernatant was transferred to clean wells, and the hemoglobin was quantified as a measure of the optical density at 405 nm (OD_405nm_) using an ELISA plate reader. RBC were treated with PBS for 2 hr or 12 hr as a negative control or 0.1% Tween 20 in water for 2 hr as a positive control. OD_405nm_ values from the positive control were used to define 100% hemolysis. Corrected values following recombinant protein treatment were calculated by subtracting the OD_405nm_ value of the PBS control at the respective timepoint. Percent lysis of the samples was calculated as 100 × (OD_405nm_ corrected sample/OD_405nm_ Tween 20). All samples were tested in triplicate in three independent experiments.

### Cytotoxicity assays

Jeg-3 cells were cultured in 48-well plates until they reached 90% confluency. Recombinant CptA protein fragments were incubated with the cells at a final concentration of 2.5 nM for 1 hr. To assess membrane integrity, media was removed and the monolayer was washed once with PBS. A 2:1 mix of PBS and 0.4% trypan blue was added to each well for 1 min, removed, and the cells were immediately imaged by light microscopy. To measure viability, 200 μl fresh growth medium and 10 μl 3-(4,5-dimethylthiazol-2-yl)-2,5-diphenyl tetrazolium bromide (MTT) were added to each well. The plates were incubated for 2 hr at 37°C in 5% CO_2_. After incubation, 100 μl isopropanol containing 0.04 N HCl was added to each well, and 200 μl of the liquid contents of each well was transferred to a 96-well plate. The optical densities at 630 nm and 570 nm were obtained using the BioTek Synergy HT, and the values for the negative control (medium plus MTT reagent and stop solution with no cells) were subtracted from the experimental values. Untreated (control) cell viability was normalized to 100% for each MTT assay.

## Results

### In silico analysis predicts structural order in the N-terminal portion of CptA

**Fig 1:**
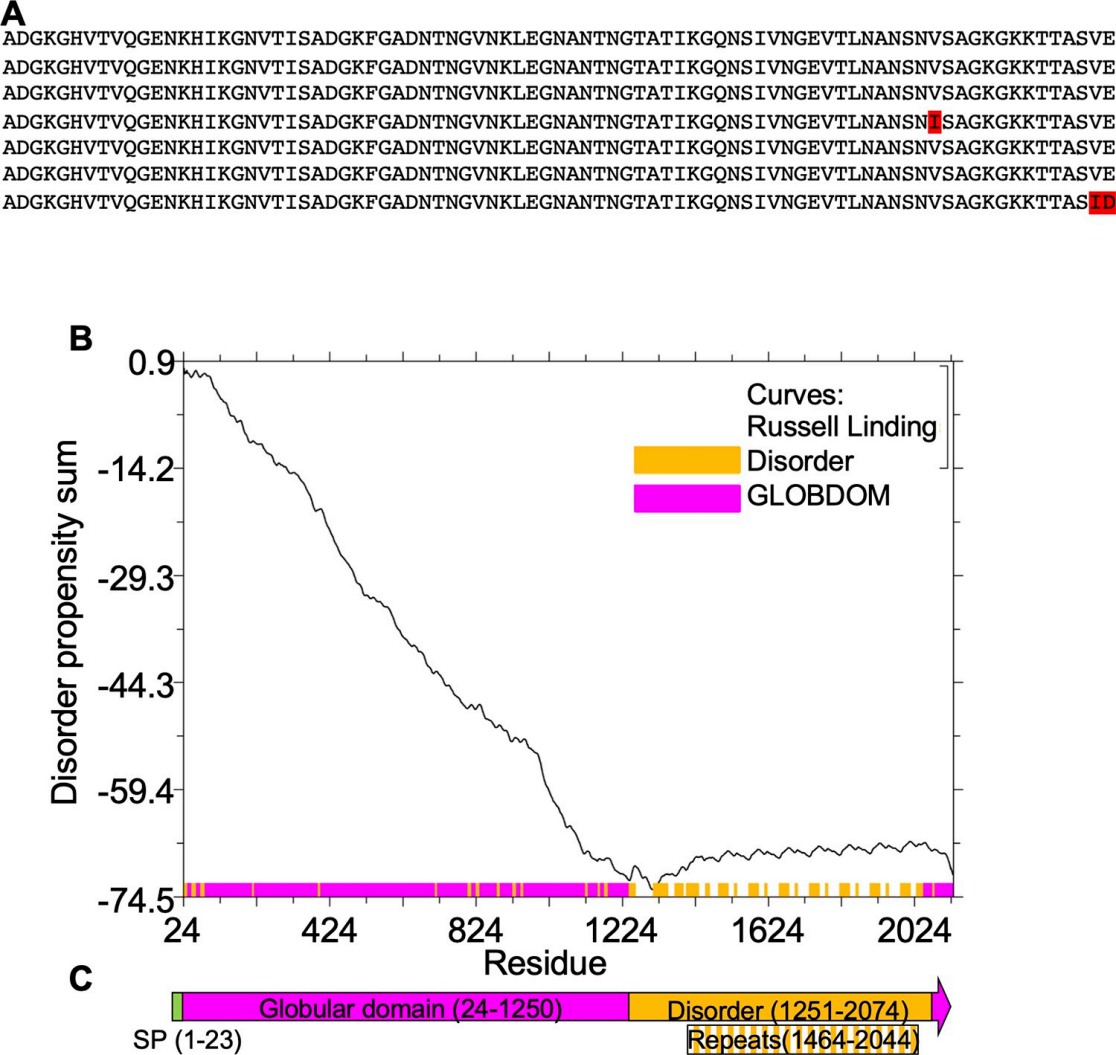
Globularity prediction within CptA. **A.** The C-terminal portion of CptA contains 7 nearly identical tandem repeats. Non-conserved residues are shown in red. **B.** The N-terminal amino acids 24–1250 of CptA were predicted by Globplot to be ordered. Amino acids 1251–2074, including the repeats from 1464–2044, were predicted to be disordered. **C.** A schematic of CptA illustrating the location of the signal peptide, globular domain, and repeat region.

CptA is a 2,130 amino acid protein with a predicted 23 amino acid SecA secretion signal at the N-terminus. It contains seven, nearly identical 83 amino acid tandem repeats near the C-terminus (Fig 1A). The CptA sequence lacking the predicted signal peptide, (amino acids 24–2,130) was analyzed by GlobPlot version 2.3 [29]. A large globular domain was predicted from amino acids 24–1250, while most of the remainder of the protein, including the repeat region, was predicted to be disordered (Fig 1B and 1C).

### A truncated form of CptA devoid of the repeat region permeabilizes Jeg-3 cells

**Fig 2:**
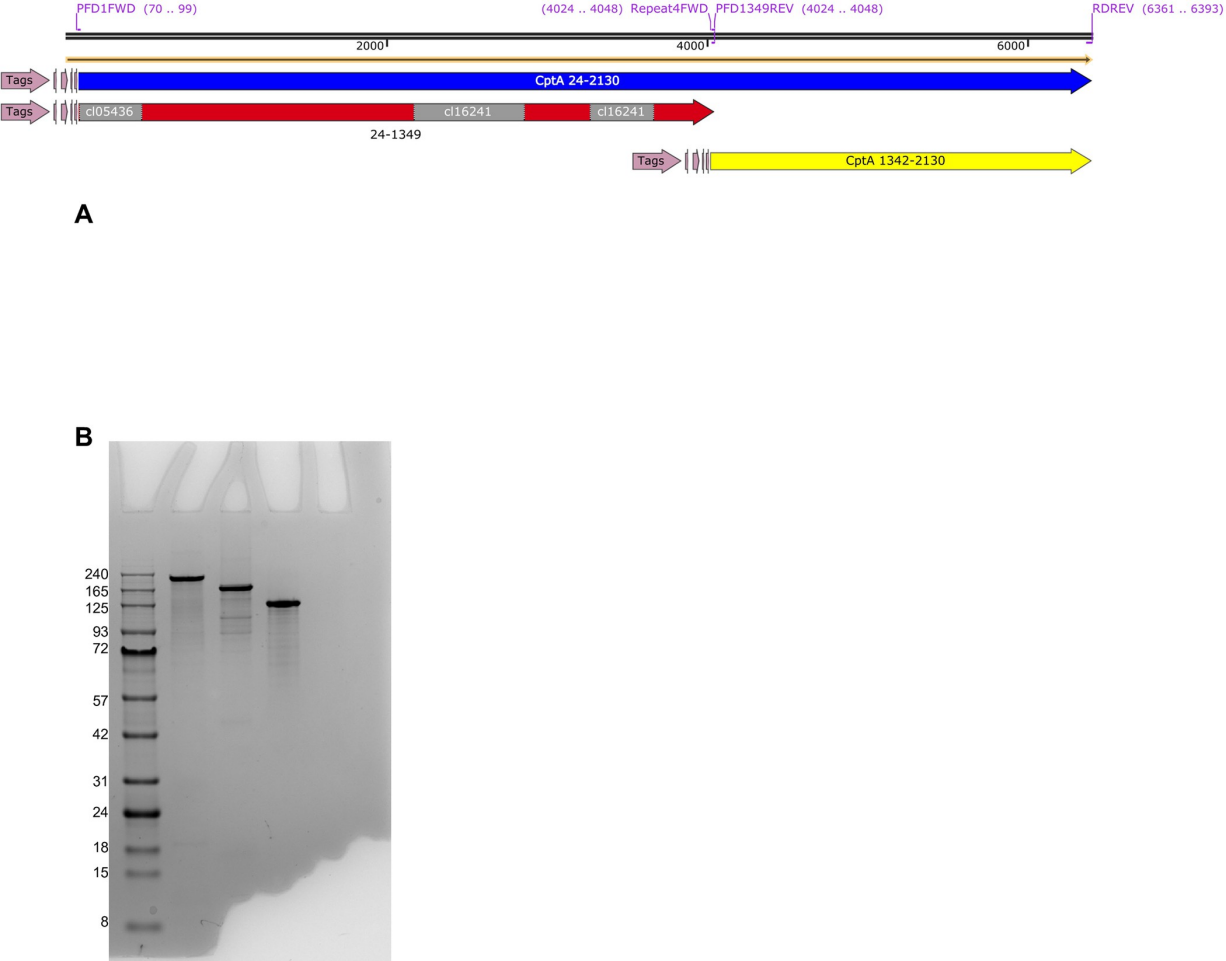
Recombinant CptA proteins. **A.** CptA1-2130 is the full-length toxin. CptA proteins that were expressed in *E*. *coli* and analyzed in this study all lacked the predicted signal peptide spanning from amino acids 1–23 (illustrations were created using SnapGene Viewer version 5.2.1). The recombinant proteins were N-terminally tagged with a Trx tag, a 6X-histidine tag, an S tag, an enterokinase cleavage site, and a thrombin cleavage site as indicated by the “Tags” region. **B.** SDS-PAGE analysis of 5 mg of each of the purified CptA proteins. Lane 1, GoldBio BLUEstain™ Protein ladder, 11–245 kDa; Lane 2, CptA24-2130, Lane 3, CptA24-1349; Lane 4, CptA1342-2130.

**Fig 3:**
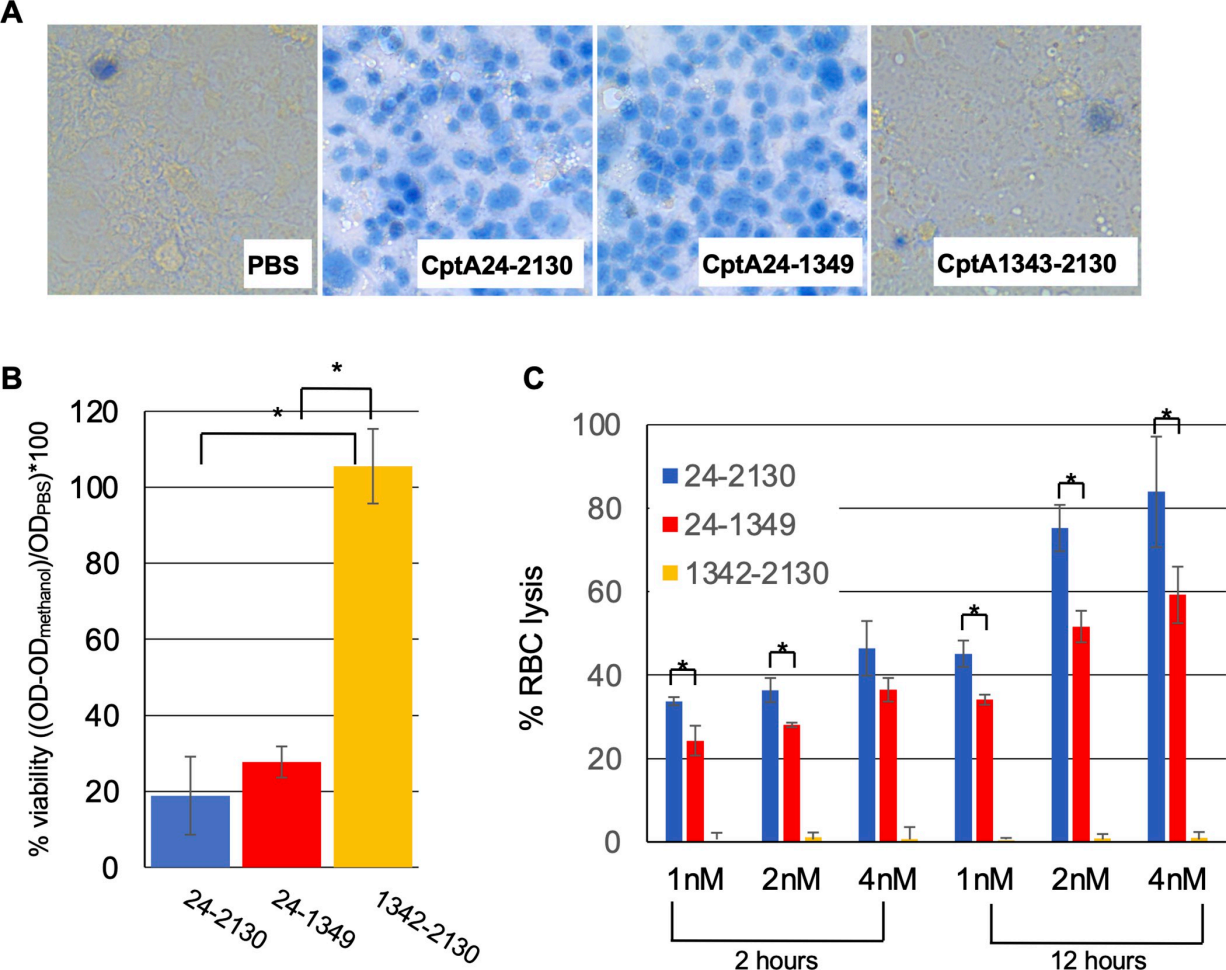
Pore-forming activity of recombinant truncated CptA. **A.** 2.5 nM of each purified recombinant protein was incubated with Jeg-3 cell monolayers for 1 hr and the treated cells were stained with trypan blue or **B.** viability was assessed by MTT assay. The y-axis represents the corrected OD of the formazan dye (OD_470nm_-OD_670nm_) minus the average corrected OD form methanol-treated cells (0% viable) divided by the average corrected OD for control (PBS) treated cells (100% viable) multiplied by 100 to produce the percent viability. **C.** For a more quantitative comparison of cell permeabilization, either 1 nM, 2 nM, or 4 nM purified recombinant protein was incubated with washed human red blood cells for 2 or 12 hours at 37°C. The reaction vessels were inverted to release hemoglobin from lysed RBC, cells were collected by centrifugation, and hemoglobin was measured by OD_405nm_. The OD_405nm_ of the mean of three replicates of vehicle control (PBS) was subtracted from the sample values and the percent hemolysis was calculated from these corrected values using the OD_405nm_ value of the positive control (0.1% Tween 20) as the standard for 100% lysis ((OD_405nm_ sample/ OD_405nm_ positive control)*100). Panel **A** shows images from single samples. These samples were analyzed in technical triplicate within each of three separate experiments. Panels **B and C** show results from triplicate technical replicates within one representative experiment. Three separate experiments were performed with similar results. For **B** and **C**, columns and error bars represent the mean and standard deviation (respectively) of the three samples from one representative experiment and * indicates statistical significance (p<0.05) by student’s t-test.

To investigate the roles of the different regions of CptA, we cloned the portion of the *cptA* gene that encodes amino acids 24 to 1349, which contains the predicted globular domain and amino acids 1342–2130, which contains the repeat region, into the pET32a expression vector and expressed the proteins CptA24-2130, CptA24-1349, and CptA1342-2130 in *E*. *coli* (Fig 2A). Purified proteins, which all included a 19 kDa N-terminal tag, were in the range of the expected molecular weights of 243 kDa for CptA24-2130, 163 kDa for CptA24-1349, and 99 kDa for CptA1342-2130 (Fig 2B). Trypan blue exclusion was used to assess the ability of the recombinant protein to permeabilize Jeg-3 cells. Similar to CptA24-2310, which comprises the full-length toxin minus the signal peptide, CptA24-1349 was able to permeabilize Jeg-3 cells to trypan blue. The repeat region, CptA1342-2130 failed to permeabilize Jeg-3 (Fig 3A). MTT assays recapitulated these findings in that CptA24-2130 and CptA 24–1349 reduced the reduction of MTT to formazan indicating that they reduced cell viability. CptA24-2130 treatment reduced viability of the cells slightly more than and equimolar concentration of CptA24-1349, but the difference was not significant (p>0.05) (Fig 3B). CptA1342-2130 did not reduce the viability of the cells. Similarly, CptA24-2130 and CptA24-1349, but not CptA1342-2130 lysed human red blood cells (Fig 3C) with the full-length toxin exhibiting significantly more hemolytic activity that the amino-terminal domain.

### Binding activity of truncated CptA proteins

**Fig 4:**
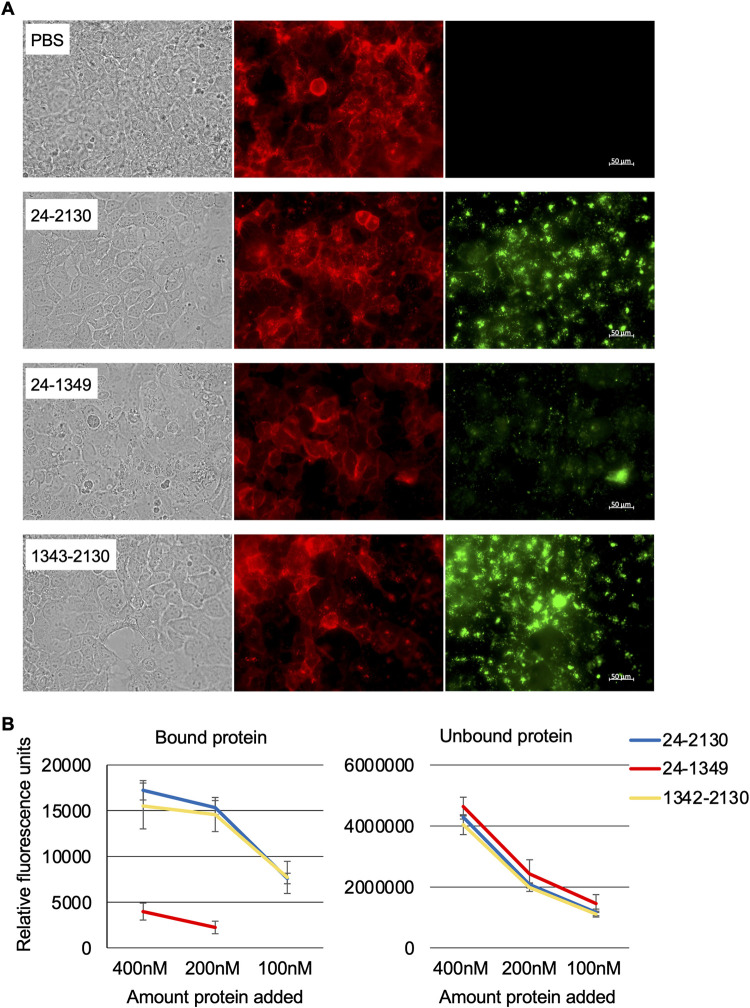
Binding of fluorescent CptA proteins to Jeg-3 cells. **A.** Microscopic examination of Jeg-3 cells treated at 37°C for 75 min with 100 nM full-length CptA24-2130, CptA24-1349, or CptA1342-2130 using immunofluorescence. Recombinant CptA and truncated proteins are visible as green and the epithelial cell membranes are red. **B.** Binding of the Alexa 488-labeled proteins was assessed quantitatively by fluorimetry.

Ostensibly, for a fragment of the CptA toxin to permeabilize epithelial cells, it would need to bind to the cells, either through a surface receptor or through its interaction with the plasma membrane. Fragments unable to permeabilize cells may or may not still have the capacity to bind. Purified, Alexa 488-labeled proteins were added to Jeg-3 monolayers and binding was examined by immunofluorescence microscopy. Microscopic examination revealed that full-length CptA, CptA24-1349, and CptA1342-2139 were all able to adhere to the surface of Jeg-3 cells (Fig 4A) although the two proteins containing the repeat region, CptA24-2130 and CptA1342-2130 adhered to a greater extent. To quantify binding, we detected fluorescence in the media (unbound protein) and in the monolayer (bound protein) of cells treated with 100 nM, 200 nM, or 400 nM of the fluorescently-labeled CptA fragments (Fig 4B). There was no significant difference (Student’s t-test p>0.05) in binding of CptA24-2130 or CptA1342-2130 when 200 nM versus 400 nM was added suggesting that binding may be saturated at 200 nM but the difference between bound CptA24-1349 at 200 nM and 400 nM was significant (p = 0.024). Binding of CptA24-1349 at 100 nM was not detectable by this method. Fluorescence of bound CptA24-2130 was 4.3-fold higher than CptA24-1349 at 400 nM and fluorescence of bound CptA1342-2130 was 3.9-fold higher than CptA24-1349 at 400 nM.

## Discussion

The association between *S*. *vaginalis* and pregnancy complications is emerging, but very little is known about the biology or virulence of this species. In this study, we initiated structure-function analysis of the CptA pore-forming toxin, currently the only virulence factor as-yet identified in *S*. *vaginalis*, through the recombinant expression of truncated proteins. The C-terminal repeat region of CptA adhered avidly to human trophoblasts but did not permeabilize them and lacked hemolytic activity. The full-length toxin bound avidly as well and was capable of permeabilizing both epithelial cells and red blood cells. The recombinant truncated toxin that included the amino-terminal globular domain but lacked the repeats, CptA24-1349, did not bind to epithelial cells as avidly as the full-length toxin or as the repeat region, but nonetheless still exhibited cytotoxic and hemolytic activities. Thus, the region between amino acids 24–1349 appears to contain the pore-forming domain and can create pores even in the absence of the C-terminal repeat region, which appears to be the major binding domain. It is possible that the 24–1349 domain contains a second binding site or that it inserts directly into the membrane without the need to a receptor.

While this study yielded insight into the pore-formation and binding domains of CptA, the role of much of this very large toxin remains unclear. There is likely a domain/s that it required for transport through the secretion partner, CptB, and this domain could be dispensable in the assays used in this study since the proteins were produced in *E*. *coli* in the absence of CptB. There could potentially be a domain involved in the acquisition of a nutrient/s that is released from host cells following pore formation. In support of this, Phyre2 [30] structural prediction modeled amino acids 36–777 of CptA with 100% confidence to the heme/hemopexin-binding protein, a two-partner system effector from *Haemophilus influenzae* (HxuA) involved in robbing heme from the host [31]. There could also be a second domain within CptA24-1349 that contributes to binding and, because pore-forming toxins often form multimers, there is potentially a domain that promotes multimerization of the toxin. Further study is needed to investigate these possibilities.

## Supporting information

S1 Raw images (JPG)
