# Supplementary figures and images for: Identification of the pore-forming and binding domains of the Sneathia vaginalis cytopathogenic toxin A
Source: PLoS One. 2023 May 4;18(5):e0284349. doi: 10.1371/journal.pone.0284349 (PMC10159106; doi:10.1371/journal.pone.0284349)

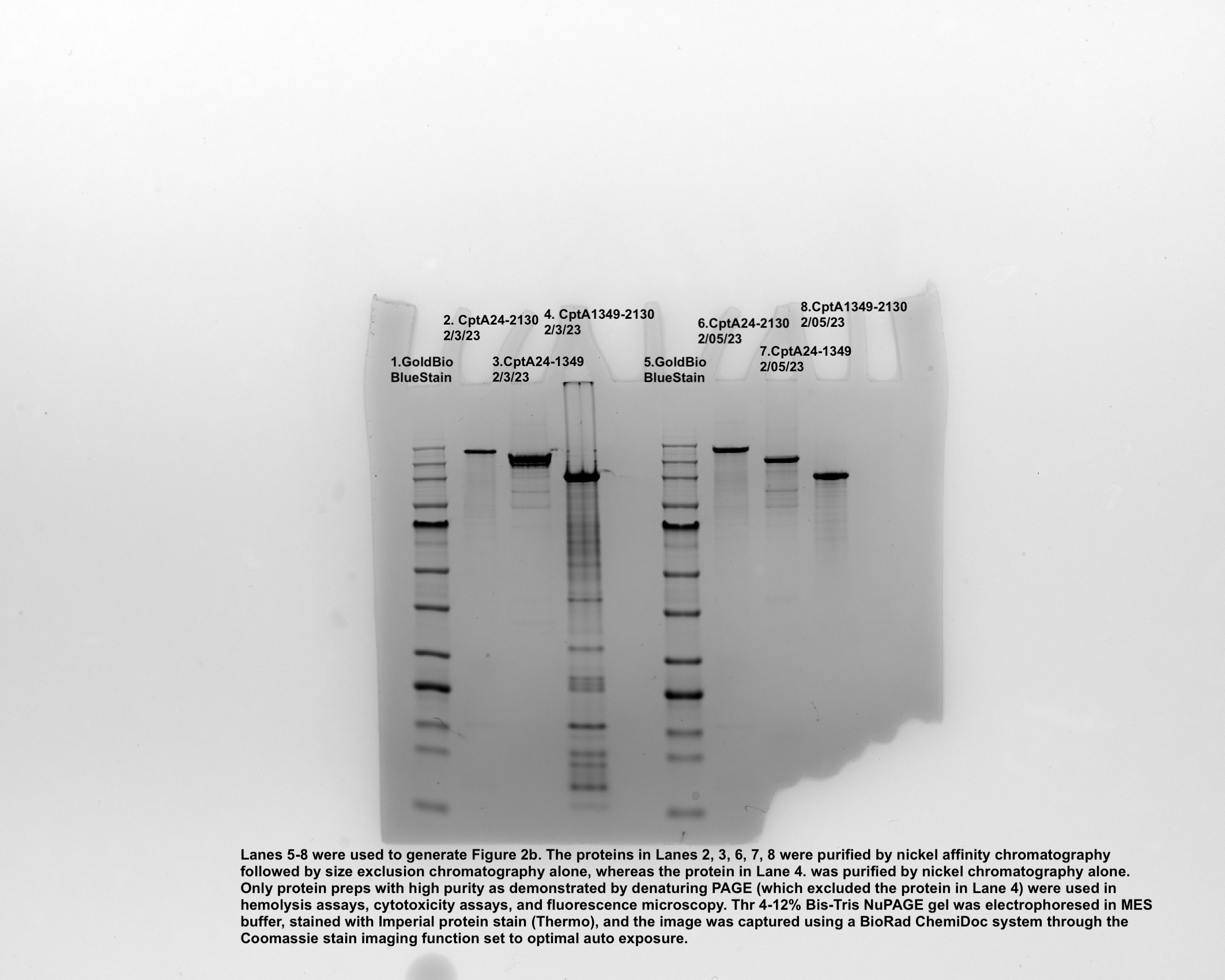

Supplement: S1 Raw images — (JPG) [file pone.0284349.s001.jpg]
